# New-onset type 1 diabetes mellitus as a delayed immune-related event after discontinuation of nivolumab: A case report

**DOI:** 10.1097/MD.0000000000030456

**Published:** 2022-09-02

**Authors:** Je Hyun Seo, Taekyu Lim, Ahrong Ham, Ye An Kim, Miji Lee

**Affiliations:** a Veterans Medical Research Institute, Veterans Health Service Medical Center, Seoul, Korea; b Division of Hematology-Oncology, Department of Internal Medicine, Veterans Health Service Medical Center, Seoul, Korea; c Division of Endocrinology, Department of Internal Medicine, Veterans Health Service Medical Center, Seoul, Korea; d Department of Pathology, Veterans Health Service Medical Center, Seoul, Korea.

**Keywords:** diabetic ketoacidosis, immune checkpoint inhibitor, nivolumab, non–small cell lung cancer, type 1 diabetes mellitus

## Abstract

Immune checkpoint inhibitors (ICIs) have revolutionized cancer treatment. However, they may cause immune-related adverse events. Although there have been a few reports of new-onset type 1 diabetes mellitus (T1DM) during ICI treatment, T1DM as a delayed immune-related event after discontinuing immunotherapy is extremely rare. Herein, we report the case of an elderly veteran who presented with diabetic ketoacidosis 4 months after the discontinuation of treatment with nivolumab.

**Patient concerns:**

A 74-year-old veteran was treated with second-line nivolumab for advanced non–small cell lung cancer. After 9 treatment cycles, the administration was discontinued due to fatigue. Four months later, he was admitted to the emergency department in a stuporous mental state and hyperglycemia, with high glycosylated hemoglobin levels (10.6%). C-peptide levels were significantly decreased, with negative islet autoantibodies.

**Diagnoses:**

We diagnosed nivolumab-induced T1DM. There were no laboratory results indicating a new thyroid dysfunction or adrenal insufficiency, which are typical endocrine adverse reactions.

**Interventions:**

Since the hypothalamic and pituitary functions were preserved and only the pancreatic endocrine capacity was impaired, we administered continuous intravenous insulin injections, with fluid and electrolyte replacement.

**Outcomes:**

His serum glucose levels decreased, and symptoms improved; hence, on the 8 day of hospitalization, we switched to multiple daily insulin injections.

**Lessons:**

The present case indicates that regular glucose monitoring and patient education are needed for diabetic ketoacidosis after the discontinuation of ICI therapy.

## 1. Introduction

Immune checkpoint inhibitors (ICIs), targeting the programmed cell death 1 (PD-1), programmed cell death ligand 1 (PD-L1), and cytotoxic T-lymphocyte antigen 4 (CTLA-4) pathway, have been a major breakthrough in the treatment of many cancer types.^[1]^ Nivolumab is a human immunoglobulin G4 PD-1 ICI antibody, which selectively blocks the PD-1 receptor on the surface of cytotoxic T cells to prevent downregulation of the immune response elicited by PD-L1 in malignant tumor cells. Nivolumab is now being frequently used, alone or in combination with chemotherapy, as the first or second line of treatment for various malignancies, including advanced non–small cell lung cancer (NSCLC).^[1]^

Although ICIs exhibit a favorable and manageable safety profile in many clinical trials, they can cause distinct toxicities known as immune-related adverse events (irAEs) as a result of enhanced T-cell activation.^[1]^ Hypophysitis and thyroid disorders are the most frequent endocrine irAEs. Moreover, diabetic ketoacidosis (DKA), as an initial presentation of type 1 diabetes mellitus (T1DM), is one of the severe and rare irAEs, occurring in less than approximately 1% of the patients undergoing ICI treatment.^[2,3]^ Recently, Couey et al^[4]^ have characterized delayed immune-related events (DIREs) posttreatment from the available data of cancer patients treated with immunotherapy. To date, few cases of T1DM have been described in patients receiving ICI therapy, while new-onset ICI-induced T1DM as DIRE after the discontinuation of immunotherapy is rare. Herein, we report a case of rapid-onset T1DM presenting with DKA, which occurred 4 months after discontinuing nivolumab in an elderly veteran with advanced NSCLC.

## 2. Case report

### 2.1 Initial diagnosis of lung cancer

**Figure 1.**
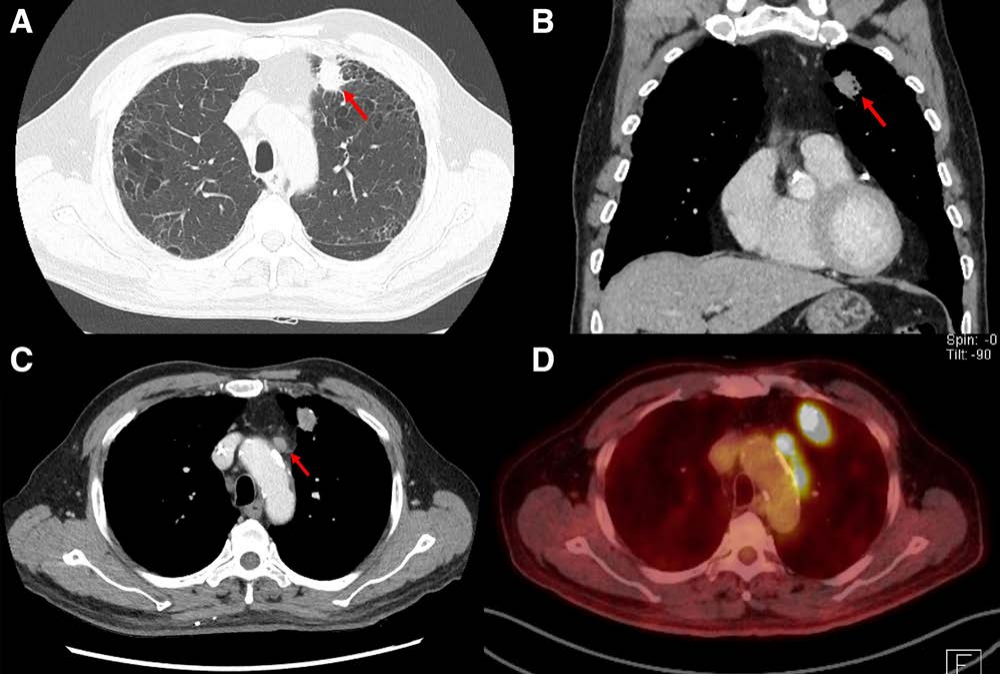
Initial diagnostic imaging data. (A) Computed tomography scan of the chest showing a 24-mm enhancing nodular lesion in the left upper lobe. (B) Multiplanar reconstruction on the coronal plane. (C) Para-aortic lymph node enlargement. (D) Positron emission tomography-computed tomographic image for para-aortic lymph node enlargement.

**Figure 2.**
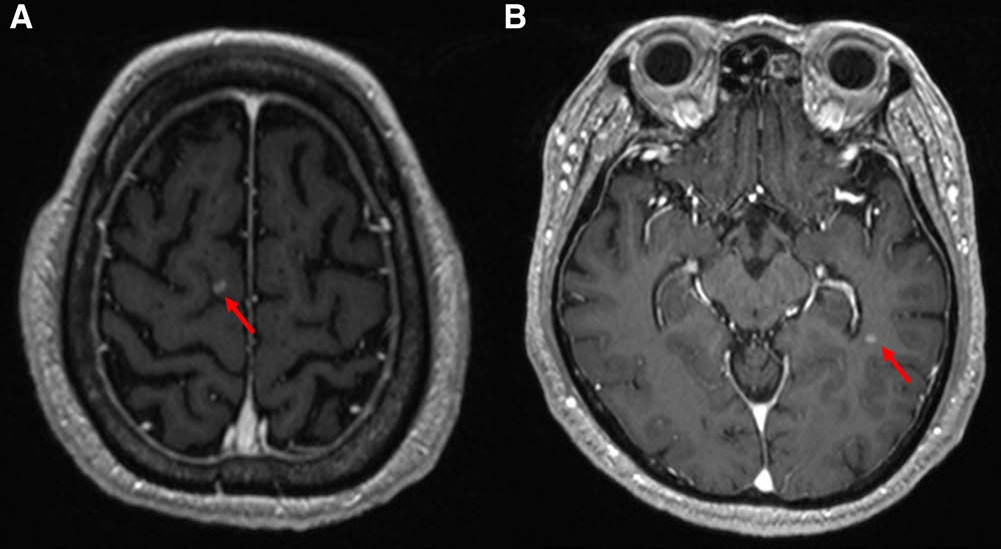
Brain MRI of the patient. T1-weighted (MPRAGE sequence showed multiple enhanced tumors at multiple sites. Arrows: Metastatic lesions in the right frontal and left temporal area. MPRAGE = magnetization prepared rapid gradient echo, MRI = magnetic resonance imaging.

**Figure 3.**
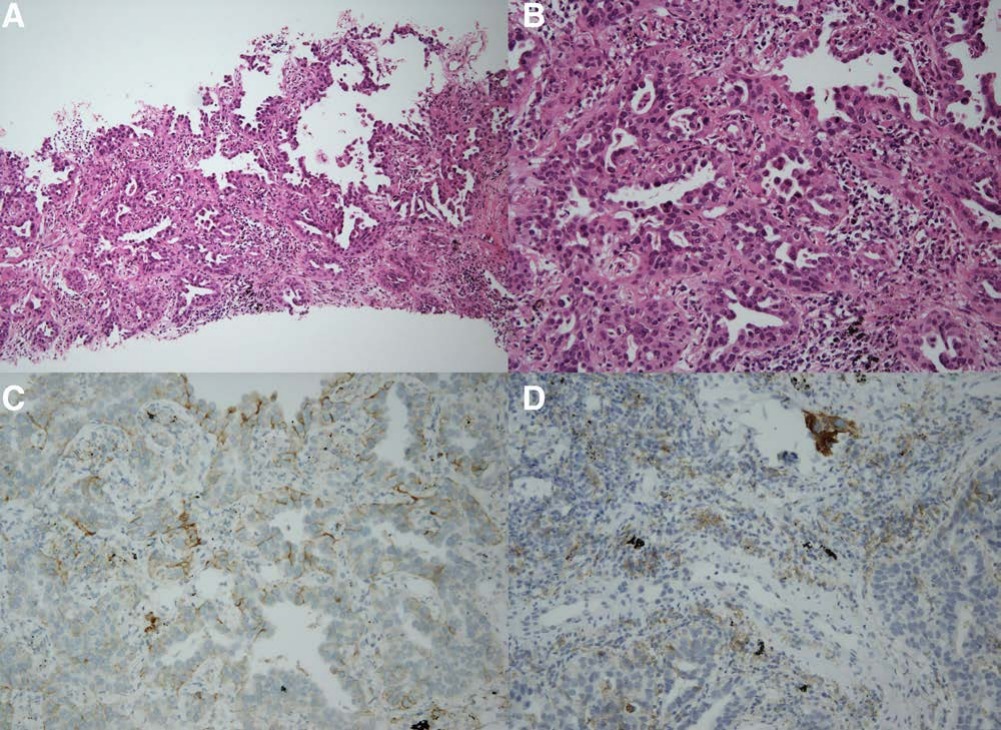
Pathology for non–small cell lung cancer from percutaneous lung biopsy. (A) H&E chemical staining (×100): H&E staining of lung demonstrated adenocarcinoma with acinar and papillary pattern; (B) H&E staining of lung (×200); (C) evaluation of PD-L1 immunostaining (SP263, ×200); and (D) evaluation of PD-L1 immunostaining (22C3, ×200). H&E = hematoxylin and eosin, PD-L1 = programmed cell death ligand 1.

In May 2019, a 74-year-old ex-smoker veteran, with interstitial lung disease and no history of diabetes, reported unexplained cough and headache. High-resolution computed tomography (CT) revealed a 2.4-cm mass in the left upper lobe. The carcinoembryonic antigen level was 6.05 ng/mL (normal, 0–5 ng/mL). In June 2019, enhanced CT manifestations of the chest revealed a 2.4-cm enhancing nodular lesion in the left upper lobe and enlarged lymph nodes in the left prevascular, para-aortic, left lower paratracheal, left hilar, and subcarinal lymph nodes (Fig. 1). A positron emission tomography-CT scan revealed significant fluorine-18-fluorodeoxy-d-glucose avidity in the primary tumor and the left paratracheal, subcarinal, hilar, and aortopulmonary window nodes, without any evidence of distant metastatic disease (Fig. 1). A brain magnetic resonance imaging revealed multiple tiny enhancing metastatic lesions in the cerebellum and bilateral frontal lobes (Fig. 2). CT-guided percutaneous lung biopsy was performed in the left lung, showing moderately differentiated adenocarcinoma, which was thyroid transcription factor 1 (+) and tumor protein 63 (–) on pathological examination (Fig. 3). In summary, the veteran was diagnosed with stage IV (cT1N1M1) NSCLC. Molecular testing showed no active *EGFR*, *ALK*, or *ROS1* mutations. Using the SP263 (Ventana) and 22C3 PharmDx (Agilent) assays, immunohistochemical staining showed PD-L1 expression with tumor proportion scores of 40% and 10%, respectively (Fig. 3).

### 2.2 Treatment course for lung cancer

**Figure 4.**
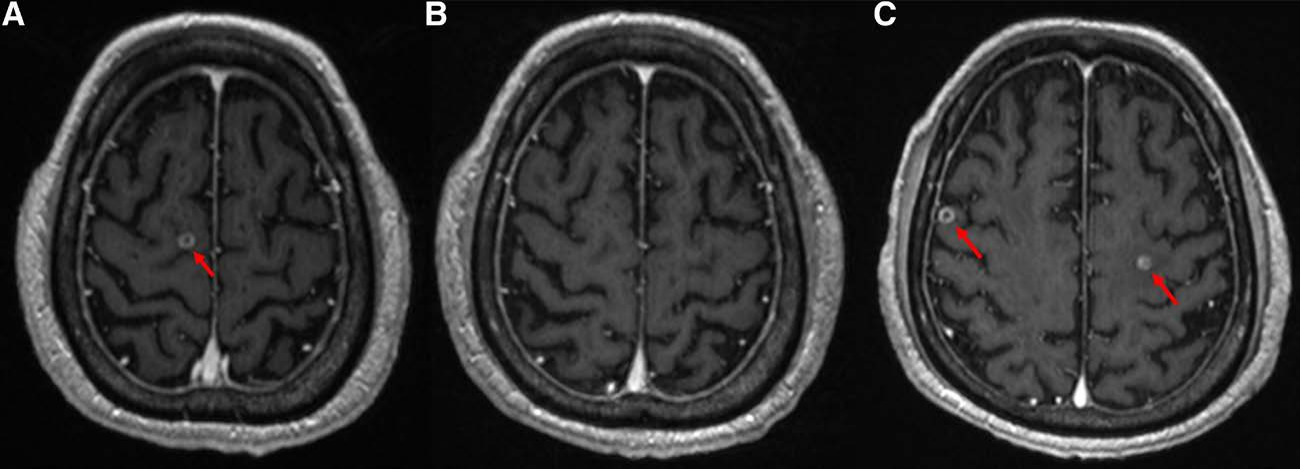
Changes in the brain MRI of the patient. (A) Baseline before WBRT on pemetrexed/cisplatin chemotherapy; (B) after WBRT: complete response status in brain; and (C) progressive disease status. MRI = magnetic resonance imaging, WBRT = whole-brain radiation therapy.

**Figure 5.**
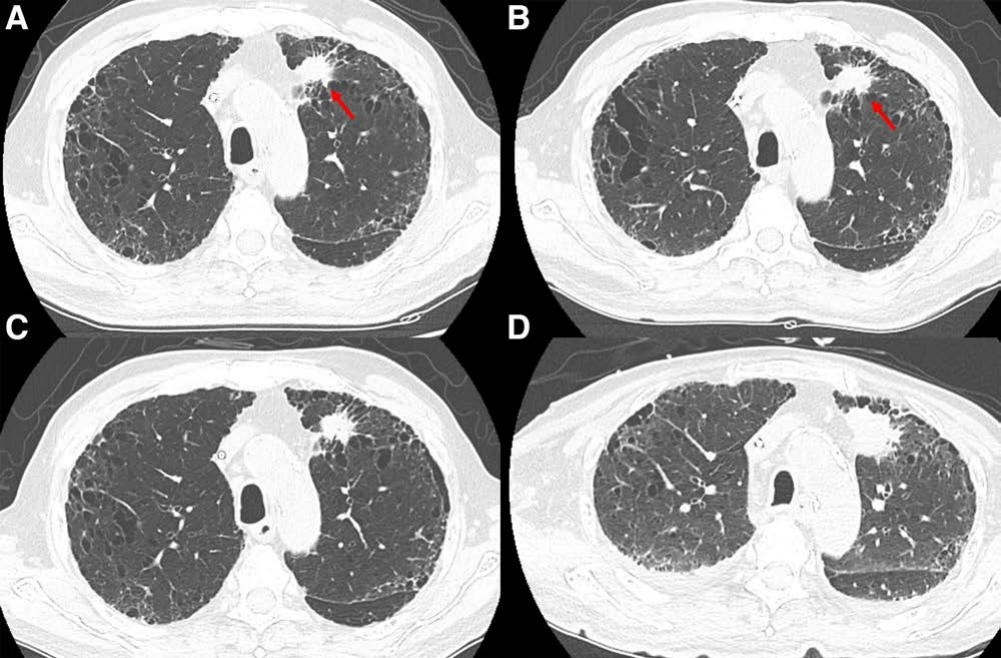
Changes in lung tumor at initial diagnosis and postchemotherapy. (A) Baseline CT scan of the chest before initiating treatment with nivolumab; (B) CT scan after 5 cycles of nivolumab (SD according to RECIST version 1.1); (C) CT scan after 9 cycles of nivolumab (SD); and (D) CT scan 4 mo after nivolumab discontinuation (progressive disease). CT = computed tomography, RECIST = Response Evaluation Criteria in Solid Tumors, SD = stable disease.

The patient was then treated with 4 cycles of pemetrexed/cisplatin followed by 9 cycles of maintenance therapy with pemetrexed administered intravenously every 3 weeks (July 2019—November 2019) and, for persistent headache, whole-brain radiation therapy with a dose of 30 Gy in 10 fractions in July 2019. According to the Response Evaluation Criteria in Solid Tumors version 1.1, partial response was achieved; however, he experienced progressive disease after 8 months (Fig. 4). The patient subsequently opted for systemic therapy using nivolumab. He received 9 cycles of nivolumab 3 mg/kg (180 mg, weight: 60 kg with BMI of 1.74) intravenously every 2 weeks (April 2020—August 2020) with stable disease according to Response Evaluation Criteria in Solid Tumors (Fig. 5). After 9 cycles, treatment was discontinued on the patient’s request, owing to fatigue and general weakness.

### 2.3 Symptom development after the discontinuation of nivolumab

Four months after the discontinuation of nivolumab, the patient suffered lower stomach discomfort, followed by dysarthria, gait disturbance, lethargy, and vomiting, 1 day before being admitted to the hospital emergency room. He was admitted in a stuporous mental state without respiratory failure. His vital signs on arrival to the emergency department were as follows: Glasgow Coma Scale score 8 (eye response 1, verbal response 2, motor response 5), blood pressure 141/114 mm Hg, heart rate 120 beats/min, respiration rate 18/min, body temperature 36.9 °C, and peripheral oxygen saturation of 97% on ambient air. Physical examination revealed normal findings except for decreased skin turgor, dry oral mucosa, and dry skin. His laboratory findings were as follows: serum glucose, 738 mg/dL; blood urea nitrogen, 39.6 mg/dL; creatinine, 2.23 mg/dL; arterial pH, 7.193; serum ketone body, 4.2 mmol/L, urine ketone body, 3+; C-peptide 0.030 ng/mL, and glycosylated hemoglobin 10.6%. Further examination revealed that the antibody to glutamic acid decarboxylase (GAD) was negative and insulin autoantibody was borderline (7.06%: normal range: 0%–7%) in Table 1. Although the chest/abdomen CT and brain magnetic resonance imaging showed worsening of previous lesions, there was neither evidence of metastasis to the pancreas nor any signs of pancreatitis. Thus, we diagnosed the patient with nivolumab-induced DKA. There were no laboratory data that suggested a new thyroid malfunction or adrenal insufficiency, which are typical endocrine irAEs. Considering that the hypothalamus and pituitary functions were intact and only the pancreatic endocrine capability was compromised, we administered continuous intravenous insulin injections, with fluid and electrolyte replacement. His serum glucose levels decreased (Table 1) and symptoms improved; hence, on the eighth day of hospitalization, we switched to multiple daily insulin injections. Following this, the patient’s systemic performance status and laboratory findings improved partially. Therefore, re-whole-brain radiation therapy was performed and hospice care was given for palliative aim.

**Table 1:** Changes of laboratory parameters related to diabetic ketoacidosis.

	Initial ER visit	HD1	HD2	HD5	HD8
Blood					
Glucose (mg/dL)	738	486	228	131	123
HbA1c (%)	10.6				
Blood urea nitrogen (mg/dL)	39.6	34.9	25.5	19.8	12.2
Creatinine (mg/dL)	2.23	1.61	1.11	0.60	0.48
Sodium (mEq/L)	135	141	150	141	137
Potassium (mEq/L)	4.9	3.4	3.2	3.4	3.3
Chloride (mEq/L)	93	112	118	105	101
Bicarbonate (mEq/L)	7.5	13.6	15.4	22.8	26.8
Anion gap	34.5	15.4	16.6	13.2	9.2
Ketone body (mmol/L)	4.2	3.4	1.2		
C-peptide (ng/mL)	0.030				
Arterial pH	7.193	7.303			
Glutamic acid decarboxylase Ab	negative				
Insulin autoantibody (%)	7.06				
Urine					
Ketone body	3+	1+	trace		

HbA1c = glycosylated hemoglobin, HD = hospital day.

## 3. Discussion

Therapy using ICIs has ushered in a new era of cancer treatment, with significant clinical outcomes observed in multiple cancers. Nivolumab is an ICI approved by the FDA in 2015 as a second-line treatment for advanced NSCLC, based on improved overall survival and a favorable safety profile in comparison to docetaxel in 2 randomized, open-label, phase III trials in advanced squamous (CheckMate 017) and nonsquamous (CheckMate 057) NSCLC with disease progression following platinum-based chemotherapy. In recently updated data of the 5-year follow-up, overall irAEs included hypothyroidism (6%) and pneumonitis (3.6%). However, no new delayed irAEs have been observed in patients who received nivolumab.^[5]^ Although neither of these studies documented any treatment-related T1DM or DKA events, <1% cases of T1DM with DKA have been reported in patients during ICI treatment.^[2,3]^ Delayed diagnosis and treatment of T1DM can be serious and life-threatening. To our knowledge, this is the first reported case to describe an elderly patient with advanced NSCLC who developed T1DM with DKA as DIRE after discontinuing nivolumab monotherapy.

ICI-induced T1DM differs considerably from conventional spontaneous T1DM. The onset age of ICI-induced T1DM is much older than that of spontaneous natural T1DM, usually seen below the age of 40 years.^[6]^ In 50% to 80% cases, ICI-induced T1DM initially presents with DKA, which frequently occurs early, within 6 weeks of initiating nivolumab treatment.^[2,3]^ In addition, low or undetectable levels of C-peptide with relatively low glycosylated hemoglobin levels are common, suggesting the rapid deterioration of β-cell function within a short duration of hyperglycemia.^[2,3]^ Furthermore, the positivity rate of islet autoantibodies (50%) is lower in ICI-induced T1DM than that in spontaneous T1DM or latent autoimmune diabetes (85%) in adults.^[3,6]^ The absence of diabetes prior to nivolumab treatment is supported by the consistently normal pre-nivolumab fasting blood glucose levels. During 1 year prior to the hyperglycemic episode, plasma glucose levels were maintained at 82 to 95 mg/dL based on 16 measurements, and the last value was 85 mg/dL. As described above, even though GAD antibodies were negative (Table 1), the inappropriately low C-peptide levels and sudden onset and persistent hyperglycemia with a presentation of DKA confirmed immune-related T1DM induced by nivolumab therapy as DIRE.

DIRE is defined as the appearance of new irAEs 90 days after the discontinuation of immunotherapy in cancer patients, which has been reported in 23 case reports. Notably, no cases of T1DM as DIRE after discontinuation of ICI therapy have been reported (Table 2). The duration of severe AE reporting following the last dose of immunotherapy was ≤ 90 days in 82% of immunotherapy clinical trials (range: 28–100 days). The median interval from postimmunotherapy to DIRE diagnosis was 6 months (range: 3–28 months) and median cumulative immunotherapy exposure was 4 doses (range: 3–42 doses).^[4]^ Chen et al have described factors such as limited follow-up periods and incomplete reporting of irAEs in a systematic review of clinical trials; hence, hurdles in assessing the true frequency and extent of DIRE are innate in current ICI clinical trials.^[7]^ In addition, Osa et al reported that the therapeutic effect may persist for at least 20 weeks after discontinuing nivolumab, since nivolumab binds to T cells for >20 weeks. Immune-mediated toxicity would be predicted to have a varied start relative to treatment, much as lasting clinical responses to immunotherapy appear to be independent of dose and treatment duration.^[8]^

**Table 2:** Characteristics of case reports of patients diagnosed with delayed immune-related T1DM.

Author (year)	Age/sex	Cancer/other irAE	Presentation	ICI regimen	Time from last ICI/Cycles	HbA1c (%)	Autoantibodies
Present case (2022)	77/ M	NSCLC/–	DKA	Nivolumab	4 mo/9	10.6	GAD (–), IAA (+)
Yaura et al (2021)^[15]^	60/F	RCC/colitis	DKA	Nivolumab + ipilimumab	6 mo/3	6.5	GAD (–)
Mae et al (2021)^[9]^	59/M	GC/–	DKA	Nivolumab	4 mo/12	10.6	GAD (–), IA-2 (–)
Nishioki et al (2020)^[10]^	73/F	NSCLC/–	DKA	Atezolizumab	4 mo/2	7.3	GAD (–), IA-2 (–), ZnT8 (–)

AAA = insulin autoantibody, DKA = diabetic ketoacidosis, F = female, GAD = glutamic acid decarboxylase, GC = gastric cancer, HbA1c = glycosylated hemoglobin, IA-2 = insulinoma-associated antigen-2, ICI = immune checkpoint inhibitor, irAE = immune-related adverse event, M = male, NSCLC = non–small cell lung cancer, RCC = renal cell carcinoma, T1DM = type 1 diabetes mellitus, ZnT8 = zinc transporter 8.

Since ICI-induced T1DM after discontinuing ICI therapy has rarely been reported, particularly in case reports, we summarize the clinical history and laboratory findings, including the present case (Table 2). Interestingly, all of these cases were negative for autoantibodies against GAD, tyrosyl phosphatase, insulin, and zinc transport, which are the main autoantibodies detected in T1DM. Patients with positive islet autoantibodies have shown a faster progression to diabetes than that in antibody-negative patients,^[3]^ which may suggest the different mechanisms of β-cell destruction in ICI-induced T1DM. Conventional T1DM is often triggered by circulating islet cell autoantibodies, while T-cell activation with altered immune response induced by ICI therapy may lead to the destruction of pancreatic β cells. Some human leukocyte antigen (HLA) profiles are associated with increased susceptibility to T1DM. Although HLA typing of our patient was not evaluated, the frequency of HLA-DR4 was reported to be proximally 40% to 60% in ICI-induced-T1DM and was much higher than that in conventional T1DM.^[3]^ Beyond HLA-risk genotype, single-nucleotide polymorphisms in PD-1/PD-L1 and CTLA-4 are potential biomarkers to predict T1DM susceptibility.^[9]^

The PD-1/PD-L1 axis is fundamental in maintaining immune homeostasis and preventing organ-specific autoimmune diseases, such as T1DM.^[10]^ The role of immune checkpoints in the pathophysiology of diabetes mellitus has been investigated in nonobese diabetic mice and in humans.^[10]^ As PD-1 interaction with its ligands, PD-L1 and PD-L2, is crucial for regulating CD4/CD8 autoreactive T cells in transgenic mice, PD-L1 expression is associated with resistance to the precipitation of autoimmune diabetes. However, nonobese diabetic mice develop rapid-onset diabetes following the blockade of the PD-1/PD-L1 axis. This corresponds with the finding that pancreatic islets express PD-L1 in mice.^[10]^ Additionally, a putative involvement of the PD-1/PD-L1 axis in T1DM pathogenesis in humans has been proposed. Both decreased PD-1 gene expression in peripheral CD4^+^ T cells and low frequency of circulating PD-1^+^ CD4^+^ T cells have been reported in patients with T1DM.^[11]^ In addition, PD-L1 is also expressed in human islet β cells with T1DM, and it is up-regulated by both type I and type II interferons via interferon regulatory factor 1, possibly to reduce the autoimmune attack.^[12]^

## 4. Conclusion and Clinical significance

ICI-induced T1DM is a rare DIRE; DKA is often the first presentation which is potentially life-threatening. With increasing number of patients being treated with ICIs, clinicians should be aware of this possible side effect. In practice, regular blood glucose monitoring and counseling on hyperglycemic symptoms, such as polyuria, polydipsia, nausea, or vomiting must be performed for new-onset T1DM with DKA and other delayed irAEs, manifesting after discontinuing ICI administration.

## Acknowledgments

The authors wish to thank Editage service for language editing.

## Author contributions

Conception and design: Taekyu Lim

Collection and assembly of data: Taekyu Lim, Ahrong Ham

Data analysis and interpretation: Taekyu Lim, Je Hyun Seo, Ahrong Ham, Ye An Kim

Manuscript writing: Taekyu Lim, Je Hyun Seo, Ahrong Ham, Miji Lee, Ye An Kim

Writing—review and editing: Taekyu Lim, Je Hyun Seo, Ahrong Ham, Ye An Kim, Miji Lee
